# Biochemical and structural characterization of the flavodoxin-like domain of the *Schizosaccharomyces japonicus* putative tRNA ^Phe^ 4-demethylwyosine synthase Tyw1 in complex with FMN

**DOI:** 10.17912/micropub.biology.000570

**Published:** 2022-06-08

**Authors:** Ljiljana Sjekloća, Adrian R. Ferré-D’Amaré

**Affiliations:** 1 Biochemistry and Biophysics Center, National Heart, Lung and Blood Institute, 50 South Drive, Bethesda, Maryland, 20892-8012, United States; 2 Current affiliation: Molecular Pathology, International Centre for Genetic Engineering and Biotechnology, Padriciano 99, Trieste 34149, Italy

## Abstract

The S-adenosyl-L-methionine-dependent tRNA 4-demethylwyosine synthase TYW1 catalyzes biosynthesis of 4-demethylwyosine (imG-14), the precursor for wyosine, the hypermodified guanine-derived nucleotide present at position 37 of phenylalanine tRNAs of archaea and eukarya. Eukaryotic TYW1 enzymes contain N-terminal flavodoxin-like and C-terminal radical-SAM domains. We determined co-crystal structures of the flavodoxin-like domain of the putative Tyw1 from *Schizosaccharomyces japonicus* in complex with flavin mononucleotide (FMN), exploiting an unexpected anomalous scatterer present in the recombinant protein. Our results show how eukaryotic TYW1 enzymes bind the coenzyme FMN and will help further elucidation of the structural enzymology of 4-demethylwyosine synthesis.

**Figure 1. Biochemical and structural characterization of fungal Tyw1 flavodoxin-like domain:**
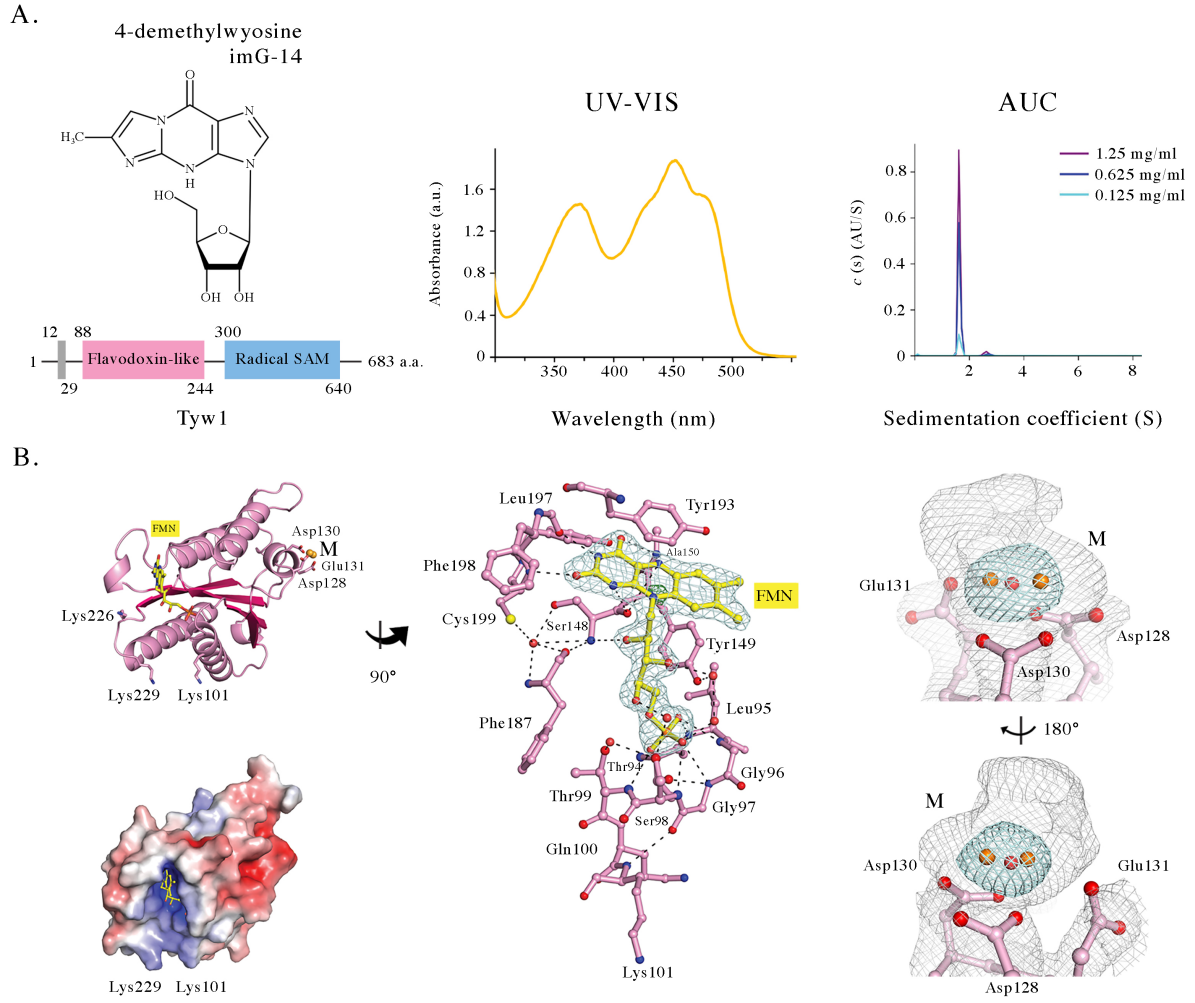
**A.** Chemical structure of 4-demethylwyosine (imG-14) and predicted domain organization of *S. japonicus* Tyw1 (grey- membrane localization sequence, pink- flavodoxin-like domain, blue- radical SAM domain); UV-VIS spectrum of purified Tyw1 76-251 in the oxidized FMN-bound form, and *c* (S) distributions derived from velocity analytical ultracentrifugation (AUC) of Tyw1 30-251, consistent with a ~23.5 kDa protein domain, in close agreement with the calculated and mass-spectrometry-derived molecular masses (24940 Da and 25010 Da, respectively). **B.** Left: cartoon representation of the crystal structure of Tyw1 76-251 with the side chains of Asp128, Asp130 and Glu131 involved in metal (M) binding, and of Lys101, Lys226 and Lys229 close to the bound FMN in stick representation, and electrostatic potential mapped onto the molecular surface of Tyw1 76-251 in the same orientation. Center: the blown-up of the FMN binding site rotated ~ 90° counterclockwise around the vertical axis, respective to the orientation shown on the left; FMN is in ball-and-stick representation, gray mesh represents |Fo| - |Fc| electron density map (contoured at 3 standard deviations above mean peak height, 3 σ) before the FMN was built in the model. After the final refinement of the model there is only a minor residual electron density around isoalloxazine nitrogen N10; residues contacting FMN (pink) and hydrogen bonds (black dashed lines), and selected water molecules (red spheres) are shown. Right: two views rotated ~180°clockwise of the portions of 2| *F*_o_ | - | *F*_c_ | (gray mesh, 1 σ) and anomalous difference Fourier syntheses (cyan mesh, 7 σ), near Asp128, Asp130 and Glu131 (ball-and-stick representation). The crystallographic model (PDB 6PUP) contains two Mn (II) ions bridged by a water molecule (orange and red spheres, respectively).

## Description

Wyosine (Y) (3-[(2 *R* ,3 *R* ,4 *S* ,5 *R* )-3,4-dihydroxy-5-(hydroxymethyl)oxolan-2-yl]-4,6-dimethylimidazo[1,2-a]purin-9-one) is a tricyclic fluorescent nucleoside introduced into archaeal and eukaryotic phenylalanine tRNA through sequential enzymatic reactions targeting G37, immediately 3' to the anticodon. In the archaeon *Methanocaldococcus jannaschii* , wyosine synthesis starts with N ^1^ -methylation of G37 by the methyltransferase TRM5 (Drogmans and Grosjean, 1987; Bjork *et al* ., 2001). Condensation of the resulting m ^1^ G with pyruvate to form the tricyclic base 4-demethylwyosine imG-14 (Figure 1A) is catalyzed by the single-domain tRNA ^Phe^ 4-demethylwyosine synthase TYW1/TAW1, a member of the S-adenosyl L- methionine (AdoMet) radical enzyme superfamily, which uses a 4Fe-4S cluster to reductively cleave SAM and activate pyruvate (Young and Bandarian, 2011; Young and Bandarian, 2015; Grell *et al* ., 2018). 4-demethylwyosine is the precursor to several wyosine derivatives detected by mass spectroscopic analysis and its hyper-modification by enzymes TYW3 and TYW4 lead to formation of wybutosine (yW) (Kasai *et al* ., 1976; Noma et al., 2006). Eukaryotic TYW1 are predicted, based on their sequences, to be multidomain, endoplasmic reticulum membrane-associated proteins (Figure 1 A Left). The best studied ortholog, *Saccharomyces cerevisiae* TYW1, comprises a membrane-spanning region followed by cytoplasmic flavodoxin-like and radical-SAM domains (Huh *et al* ., 2003; Waas *et al* ., 2005; Noma *et al.* , 2006; Li *et al* , 2011). Wyosine and its derivatives may modulate translation efficiency by tuning the affinity of tRNA ^Phe^ for phenylalanyl-tRNA synthetase (Krauss *et al* ., 1976) and by selectively preventing frameshifting (Hatfiled *et al.* , 1989; Noma *et al.,* 2006; Helm and Alfonzo, 2014). The *S. cerevisiae* Δ *tyw1* strain shows no phenotypic changes. However, reduced growth resulting from deletion of the iron importer protein gene *CCC1* can be overcome by TYW1 overexpression, suggesting TYW1 involvement in regulation of cytoplasmic free iron (Li *et al.* , 2011).

As an initial step in elucidating the role of flavin mononucleotide FMN in wyosine biosynthesis, we determined crystal structures at 1.5 Å and 1.9 Å-resolution of the flavodoxin-like domain of the putative *Schizosaccharomyces* *japonicus* Tyw1 in complex with FMN. *S. japonicus* Tyw1 recombinant fragments 30-251 and 76-251 were expressed in aerobically grown *E. coli* . The purified proteins are monomeric, and their intense yellow color and UV-VIS spectra are consistent with the presence of oxidized flavin mononucleotide FMN _(ox)_ (Figure 1A Center). Diffraction data from Tyw1 76-251 crystals collected using 1 Å X-radiation extended to 1.9 Å resolution. Data analysis revealed anomalous signal, which was employed for structure determination by the single-wavelength anomalous dispersion (SAD) method (PDB 6PUP). Diffraction data from Tyw1 30-251 crystals collected using 1.1 Å X-radiation extended to 1.5 Å resolution and did not exhibit anomalous signal. This structure (PDB 6PUQ) was solved by molecular replacement using the Tyw1 76-251 structure (PDB 6PUP) as a search model. In the 6PUQ structure, there was no electron density corresponding to residues 30-80, suggesting disorder.

Tyw1 residues 81-250 adopt a Rossman-like fold comprised of a parallel, 5-stranded β-sheet flanked by α-helices, two on each side (Figure 1B Left, Extended Data Figure 1). The β-strand connectivity (2, 1, 3, 4, 5) is typical of flavodoxins, and the last strand (β5) does not contain an insertion (Extended Data Figure 2). Hence Tyw1 81-250 is a short-chain, flavodoxin-like domain (Drennan *et al.* , 1999). Loops at the N-terminal ends of the β-strands create a binding site for a single FMN molecule (Figure 1B Center). The isoalloxazine is in a planar conformation (Figure 1B Center). The pyrimidine ring hydrogen bonds with the backbone Cys199 and Leu197. The phenilenediamine ring forms polar contacts with the Ser148 carbonyl; this ring is shielded from solvent on its *si* face by Tyr149 and on *re* face by Tyr193 with which it makes π-π stack. The dimethylbenzene ring is the only solvent-exposed portion of the isoalloxazine. The ribityl oxygens are hydrogen-bonded to the Ser148 amide, and to two water molecules which in turn H-bond with Cys199 and Tyr149. The FMN phosphate-binding loop (Thr94-Leu95-Gly96-Gly97-Ser98-Thr99) conforms to the phosphate binding Pi-loop consensus motif T/S-X-T-G-X-T (Drennan *et al.,* 1999), except for the glycine in the third position. In addition, the Gly97 carbonyl makes polar contact with the Lys101 amide. Lys101, Lys226, and Lys229 flank a ~8 Å-wide entry point to FMN- binding site, which might be relevant FMN binding, and, in the context of the full length Tyw1, for access to tRNA ^Phe^ m ^1^ G37 and pyruvate.

Diametrically opposite the FMN binding site there is a system of several loops (Figure 1B Left), the longest of which (residues 168-180) contains Asp168-Phe169-Arg170 and Leu176, residues conserved in all TYW1 proteins (Extended Data Figure 2). This loop is the most structurally dissimilar from the nearest structural homologues identified by a DALI search: eukaryotic NADP(H)-cytochrome 450 diflavin oxidoreductase (3QFC) and nitric-oxide synthase (1TLL), and bacterial flavodoxin (5LJI) and sulfite reductase NADP(H) flavoprotein (1YKG) (Extended Data Figure 2). The unexpected anomalous feature present in our Tyw1 76-251 crystals, which is within coordination distance of Asp128, Asp130 and Glu131, was modeled as two Mn ^+2^ ions bridged by a water molecule (Figure 1B Right) based on the strength and shape of the anomalous signal, the coordination geometry of the observed binding site, and manganese bioavailability in the expression host. The absence of these bound Mn ^+2^ ions in our Tyw1 30-251 crystals might be due to use of a different recombinant tag, occlusion of the binding site by the flexible amino-terminal extension Tyw1 30-80, different crystallization conditions.

In summary, our experimental observations prove that *Schizosaccharomyces japonicus* Tyw1 has a stable, flavodoxin-like domain capable of recognizing FMN. Flavodoxin-like domains are found in proteins that function in electron transport systems (oxidoreductases, transferases, lyases, isomerases, and ligases) and the presence of a possible functional metal ion-binding site in Tyw1 flavodoxin-like domain, together with binding of an iron-sulfur cluster in the Tyw1 carboxyl terminal domain, might contribute to Tyw1 functions. Structural similarity of its flavodoxin-like domain to those of enzymes that couple NADP(H) and FAD/FMN for their activity suggests regulation of Tyw1 by the cellular redox state. The interplay of flavin and NADH/NADPH in TYW1 proteins was also noted by Young and Bandarian (Young and Bandarian, 2021) after the atomic coordinates for our structures were released. These results will help further elucidation of the structural enzymology of 4-demethylwyosine synthesis in eukaryotes.

## Methods

*Schizosaccharomyces japonicus* yFS275/FY16936 *tyw1* fragments encompassing Tyw1 residues 30-251 and 76-251 were cloned in bacterial expression vectors conferring N-terminal TRX-6His or MBP-6His tags and a TEV site. Cloning details are summarized in Extended Data Table 1. Transformed *Escherichia coli* KRX (Promega) were grown in Terrific Broth medium supplemented with 100 µg l ^-1^ ampicillin at 310 K until OD _600_ ~ 0.6 and recombinant protein expression was induced with 1 mM IPTG and 0.2 % rhamnose for 12 hr at 291 K. Cells were lysed in 0.5 M NaCl, 20 mM imidazole, 5% glycerol, 50 mM Hepes-NaOH pH 7.8, 0.5 mM 2-mercaptoethanol (LyB buffer) containing 1 mM PMSF. Recombinant proteins were applied on nickel nitrilotriacetic acid affinity (NiNTA) resin (Qiagen) and eluted in LyB supplemented with 330 mM imidazole, exchanged in LyB on 10 kDa cut-off concentrator (PALL Corporation) and digested with 6His-tagged TEV, passed again over NiNTA, concentrated, and loaded on HiLoad Superdex 75 pg 16/60 column (GE Healthcare) in 0.5 M KCl, 20 mM NaCl, 5% glycerol, 2 mM MgCl _2_ , 50 mM Hepes-KOH 7.5, 0.5 mM 2- mercaptoethanol, and exchanged into 150 mM KCl, 20 mM NaCl, 2 mM MgCl _2_ , 5% glycerol, 50 mM Hepes-NaOH pH 7.5, 0.5 mM 2-mercaptoethanol (AUC buffer). Analytical ultracentrifugation was performed in AUC buffer, at 293 K, absorbance was measured at 280 nm, run speed was 60,000 RPM, and the reference cell contained AUC buffer. Data were analyzed using SEDFIT (Schuck and Rossmanith, 2000), assuming a partial specific volume of 0.73 cm ^3^ g ^-1^ and hydration value of 0.3.

*S. japonicus* Tyw1 constructs were crystallized by vapor diffusion. Hanging drops were prepared by mixing 200 nl of protein and 100 nl of reservoir solution using a Mosquito robot (TTP LabTech) and equilibrated against 100 µl of reservoir solution. *S. japonicus* Tyw1 76-251 (concentrated to ~38 g l ^-1^ ) crystallized as yellow tetragonal prisms at 295 K using a reservoir solution comprised of 100 mM Hepes-NaOH pH 7.5, 100 mM MOPS pH 7.5, 9.4% (v/v) MPD, 9.4% (v/v) PEG1000, 9.4% (w/v) PEG 3350, 300 mM MgCl _2_ , and 300 mM CaCl _2_ . Crystallization details are summarized in Extended Data Table 2. Crystals grew to maximal dimensions of 150 x 50 x 50 µm ^3^ in 10 days. *S. japonicus* Tyw1 30-251 (concentrated to ~12 g l ^-1^ ) crystallized as yellow tetragonal pyramids crystals at 288 K using a reservoir solution comprised of 200 mM sodium acetate, 30% (w/v) PEG 4000, 100 mM Tris- HCl pH 8.5. Crystals grew to maximum dimensions of 300 x 300 x 300 µm ^3^ within two weeks. Crystals were transferred to their respective reservoir solutions supplemented with 20% (v/v) PEG 400, mounted in nylon loops, and flash-frozen by plunging into liquid nitrogen. After exposure to X- rays, crystals were still yellow, and no color alteration was visible.

Diffraction data were collected at 100 K in rotation mode at beam lines 5.0.1 and 5.0.2 of the Advanced Light Source, Lawrence Berkeley National Laboratory (ALS) using 1.0 Å X-radiation, and integrated and scaled with DIALS (Winter *et al* ., 2018). Data collection and processing statistics are summarized in Extended Data Table 3.

The Tyw1 76-251 (PDB 6PUP) structure was solved by single-wavelength anomalous dispersion (SAD) and automatically built using the Crank2 pipeline implemented in ccp4i2 (Skubak *et al* ., 2018; Potterton *et al* ., 2018). The mean overall figure of merit prior to density modification was 0.148. The model was manually rebuilt (Emsley and Cowtan, 2004) and was refined using Refmac5 (Murshudov *et al* ., 2011). The final refinement statistics are summarized in Extended Data Table 4. Although the identity of the anomalous scatterer in the Tyw1 76-251 fragment is not certain (see below), it was modeled as a binuclear, water bridged Mn ^+2^ complex, based on anomalous scattering, B-factor and geometric analysis. Tyw1 30-251 data did not exhibit anomalous signal, and its structure (PDB 6PUQ) was determined by molecular replacement (McCoy *et al* ., 2007) using the Tyw1 76-251 structure (6PUP) as search model. The top solution had LLG and TFZ scores of 960 and 28.8, respectively. The model was refined using Phenix-Refine (Adams *et al* ., 2010) (Extended Data Table 4). FMN density was strong and was modeled with unit occupancy in both structures. Structural figures were generated with PyMol (DeLano, 2002).

## Reagents

*Schizosaccharomyces japonicus* *tyw1* Uniprot B6K6D6_SCHJY strain yFS275/FY16936 synthetic gene
